# Recognising and healing emotional wounds of child labourers: call to action based on the evidence and stakeholder views from India and Nepal

**DOI:** 10.1192/bji.2021.50

**Published:** 2022-05

**Authors:** Harleen Kaur, Kathleen Duncan, Sandesh Dhakal, Narayan Sharma, Shanta Niraula, Rakesh Pandey, Veena Kumari, Jennifer Y. F. Lau, Tushar Singh

**Affiliations:** 1 MSc, Research Scholar, Department of Psychology, Banaras Hindu University, Varanasi, India; 2 Student, Division of Psychology & Language Sciences, University College, London, UK; 3 PhD, Lecturer, Central Department of Psychology, Tribhuvan University, Kathmandu, Nepal; 4 PhD, Lecturer and Head of the Department, Department of Philosophy and Psychology, Tri-Chandra Campus, Ghantaghar, Kathmandu Nepal; 5 PhD, Former Head, Central Department of Psychology, Tribhuwan University, Nepal; 6 PhD, Professor, Department of Psychology, Banaras Hindu University, Varanasi, India; 7 PhD, Professor, Centre for Cognitive Neuroscience, College of Health, Medicine and Life Sciences, Brunel University London, UK; 8 PhD, Professor of Youth Resilience, Youth Resilience Research Unit, Wolfson Institute of Population Health, Queen Mary University of London, UK; 9 DPhil, Assistant Professor, Department of Psychology, Banaras Hindu University, Varanasi, India. Email: tusharsinghalld@gmail.com

**Keywords:** Child labourers, mental health, mental health interventions, care providers, psychoeducation

## Abstract

Child labourers are at risk of poorer mental health and once rescued require urgent mental health interventions to ameliorate the long-term impact. In our review, only two published scientific studies evaluated custom-made interventions; other programmes were obtained from non-governmental organisations (NGOs), which need rigorous trial evaluation. We also sought the viewpoints of stakeholders working directly with rescued young people, as well as consulting young people with lived experiences of child labour. We propose that psychoeducational interventions aimed at employees working directly with young people could represent a fruitful approach for low- and middle-income countries in the Asia-Pacific region but also more generally.

Child labour, a pressing human rights issue, is prevalent in many Asian low- and middle-income countries (LMICs), with a report released in 2017 by the International Labour Organization (ILO) citing around 1 in 14 young people employed illegally in the Asia-Pacific region.^1^ Child labour is defined by the ILO on the basis of its harmful consequences, most notably, its interference with schooling. Indeed, data show the impact of child labour on school attendance and educational attainment,^2^ which in turn contributes to the intergenerational transmission of poverty within families^3^ and reduced accumulation of human capital for economies. Eliminating child labour is therefore an increasing focus of intergovernmental and non-governmental organisations (NGOs) but until these efforts completely succeed, the provision of support to young people currently working illegally or those who have been rescued is an urgent practical task. Indeed, child labour involves work that is ‘mentally, physically, socially or morally dangerous and harmful to children’.^4^ Recently, we also uncovered widespread experience of maltreatment among rescued adolescent labourers in India and Nepal. Using the Juvenile Victimization Questionnaire (JVQ),^5^ a large proportion of each sample reported exposure to childhood abuse and/or neglect (83.36% in the India sample, 72% in the Nepal sample). The most common experiences were physical abuse (reported by 46.6–72.7%) and emotional abuse ((40.8–47.7%).^6,7^ These experiences were associated with poor mental health, particularly symptoms of affective disorders,^6,7^ consistent with other published scientific studies.^8–13^ Speaking more directly to these points, our research team also consulted with six care-home employees (who had between 2 and 11 years of experience working directly with young labourers in India and Nepal) both with and without social work or counselling backgrounds. The sample comprised stakeholder consultants comprised both front-line care staff and those in management positions, including the founder of an NGO. These consultations also firmly indicated that mental health interventions were an urgent priority. One social worker in a care-home in India said:

‘Mental health is utmost important, as I told you earlier also, the capacity of a child to think, to understand someone or some situation has completely vanished […] They have printed in their thinking that they are born to be exploited, they can only experience the emotions of fear and sadness, so it is really very essential for them to understand there lies another world of which they are not aware yet […] we need to work a lot on their mental health.’

As early anxiety and mood symptoms are likely to have an impact on other key outcomes in youth (e.g. education),^11,12^ intervening to target these can help to achieve other goals of rehabilitation (such as catching up with schoolwork, developing vocational interests, hope for the future).

## The evidence base for interventions

### Randomised controlled trials

Although there are interventions aimed at preventing childhood abuse, including strategies to detect maltreatment of children and youth, there have been few ‘custom-made interventions’ specifically targeting mental health problems in young people with a history of child labour, and reviews of this limited literature are also lacking.^14^ When assessing mental health interventions in children and young people who have been exposed to likely exploitation (e.g. street children, child soldiers), only two relevant studies emerged.

A randomised controlled trial by McMullen and colleagues^15^ evaluated a 15-session, culturally adapted, group-based trauma-focused cognitive–behavioural therapy (TF-CBT) in 39 male Ugandan former child soldiers and also 11 war-affected boys. Results showed a reduction of psychosocial distress and symptoms of post-traumatic stress disorder, mood and anxiety disorders and conduct problems, with a significant increase in prosocial behaviour, compared with the waiting-list control group.

In a second study that involved a partnering NGO (Rukha), Hoffman and colleagues^16^ measured the impact of an intervention on mental health problems in 107 Brazilian youth street workers. The intervention involved parallel financial and psychosocial strategies; children were required to cease street work while being provided with a monthly stipend. Families were offered constructive strategies on how parents could become more involved in their children's educational needs and life skills, and how to engage in more open forms of communication within the family. At 2-year follow-up, mental health difficulties were significantly lower than at baseline, yet over 50% of children included in the study still reported mental health problems, with those showing sustained difficulties being predicted by higher levels of caregiver mental health problems, poorer family relationships, higher poverty and reported neglect at baseline.^16^

While street children and child soldiers have different experiences to child labourers, they may share experiences of exploitation by adults and an assumption of adult roles, with all groups being exposed (albeit to differing degrees) to trauma and violence. Thus, these few studies evaluating mental health interventions in vulnerable youth groups have some potential for extension to child labourers. However, across studies, there remain methodological limitations, such as in the nature of the control groups (or their absence) and the lack of standardised measurement tools to track symptom changes, as well as conceptual ones, such as poor understanding of the hypothesised mechanism of action. To address the paucity of mental health interventions tailored to the specific needs of young labourers, our group has begun to develop and formatively evaluate computerised interventions targeting cognitive vulnerability factors associated with anxiety and depression in rescued child labourers in India and Nepal (ClinicalTrials.gov identifier: NCT03625206, date of registration: 10 August 2018).

### NGO reports and our own consultations

Another rich source of information about potential interventions that are developed specifically for child and adolescent labourers comes from reports published by NGOs. Intervention delivery draws on the rich experiences of these stakeholders but the interventions offered may not yet have been subjected to rigorous evaluation frameworks guiding their development. The NGO Global March Against Child Labour creates children's groups in schools to increase former child laborers’ participation in education, feelings of connectedness, engagement in age-appropriate activities and psychosocial well-being.^17^ Similarly, in their CLEAR campaign, the NGO ECLT Foundation (Eliminating Child Labor in Tobacco Growing) has created ‘youth groups’ for sharing experiences, operating alongside ‘mother groups’ that provide psychosocial support to increase social well-being, playtime, pride, confidence, happiness, and optimism about the future.^18^ Save the Children has offered the programme ‘Healing and Education through Arts’ (HEART) to over 350 000 children in 22 countries.^19^ HEART provides children affected by chronic stress the opportunity to ‘share memories through artistic expression’, as well as ‘increase social and emotional wellbeing, educational experiences, and feelings of connectivity to peers and adults.’^19^

Some of these initiatives mirror the strategies and activities used frequently by those working directly with young people to help them manage their emotions and improve their well-being. Again, our consultations with care-home employees were insightful, highlighting possible research initiatives in the co-design of interventions for these vulnerable young people. One care-home employee talked about strategies for increasing self-compassion among her clients, such as:

‘Telling oneself daily, say to yourself that I love myself, one small activity we do is coming in front of a mirror and start saying positive words, like I am strong, I am blessed, I can do it, etc.’

and potentially embedding this within creative representations and activities:

‘Some children like drawing so we ask them to draw, some make a sun so we teach them you are as bright as a sun is, some make stars so we say you will rise and shine like a star […] If someone is good at music or likes music, we write a motivating song and give it to them so that lyrics, that music motivates them, and they like doing that activity.’

Another mentioned practising ‘life skills’, such as ‘exercising, praying and meditating daily’. Finally, one stakeholder (the founder of an NGO) talked about developing vocational skills and identifying role models to help motivate young people to define their future career aspirations.

In addition, we also sought the views of seven young people, currently in out-of-home care after being rescued from illegal labour. Four of these seven agreed that ‘feeling well’ was quite or very important to them and that they would be quite or very motivated to attend sessions that helped them to ‘feel better’. Among the different options for interventions, young people welcomed equally choices between face-to-face therapy sessions with counsellors and peer-to-peer group support sessions. Young people were also receptive to daily mindfulness and meditation exercises to learn to control distracting thoughts and the use of art and creative activities to explore their emotions and thoughts. Only one of the seven endorsed the idea of using technology such as a computer for learning how to regulate their emotions.

## The future

Leveraging the valuable insights from these NGO-driven programmes, the expertise of stakeholders such as teachers, social workers and counsellors, and young people themselves within co-designed interventions should be an important next step in intervention development. These should then be formatively evaluated for feasibility and acceptability before refining. However, while these collaborative initiatives are being pursued, there also remain challenges of under-recognition of mental health problems by stakeholders working with these young people. This was evident in those in positions of senior management (e.g. the founder of an NGO in India said: ‘Although mental health needs are important, I haven't found any issue related to mental health with at least these 200–250 children I have dealt with so far’) as well as those who directly care for young people but who may not have been trained in child psychology. Indeed, consultations with ‘care-home parents’ in an NGO in Nepal, who often provide for the needs of these young people, indicated that they would value training in better understanding and recognition of the mental health needs of their client group. Finally, young people did not always seem to value the importance of ‘feeling well’, with three of the seven young people in our consultation saying that this was only ‘a little important’ to them; this suggests that there is a stigma associated with discussing mental health and well-being, possibly fuelled by poor understanding.

We therefore call for the development of a two-pronged approach to interventions to help child labourers in LMICs in the Asia-Pacific region and more generally: (a) educational interventions aimed at care-home employees to better recognise mental health problems (particularly affective disorders) in their clients and increase their capacity to support and encourage young people to seek further assessment; and treatment and (b) mental health interventions aimed at and co-produced with young people to empower them to better manage emerging mood and anxiety symptoms.

## Data Availability

The data that support the findings of this study are available from the corresponding author, [TS], upon reasonable request.
